# SUV driving “masculinizes” risk behavior in females: a public health challenge

**DOI:** 10.1007/s00508-017-1219-6

**Published:** 2017-06-02

**Authors:** Peter Wallner, Anna Wanka, Hans-Peter Hutter

**Affiliations:** 1 Institute of Environmental Health, Center for Public Health, Medical University Vienna, Kinderspitalgasse 15, 1090 Vienna, Austria; 2 Institute of Sociology, University of Vienna, Vienna, Austria

**Keywords:** Observational study, Public health, Gender, Road safety, Traffic violation

## Abstract

Involvement of sport utility vehicles (SUV) in accidents especially with children is of increasing importance. Studies have indicated a more risky behavior in SUV drivers. We conducted an observational study focusing on traffic violations, car type, and the gender of the driver in Vienna. The study was conducted on five weekdays at the beginning of school term. Three busy intersections were selected.

Drivers of 43,168 normal cars and 5653 SUVs were counted at the intersections during the observation period. In total 13.8% drivers were unbelted, 3.1% were using a handheld mobile phone, and 2.5% violated traffic lights. These frequencies were significantly higher in SUV drivers than in normal passenger car drivers. This “SUV effect” also occurred in women for all violations, although male drivers violated traffic laws more often than female drivers. However, for driving unbelted the difference between males and females was smaller in SUV drivers.

## Introduction

Human behavioral factors including use of a handheld mobile telephone while driving and not using seat belts increase risk of car accidents or severity of crash injuries [1–3]. However, despite legal regulations including sanctions, noncompliance is high [4, 5].

These habits have been linked to situational, legal, social, and personality factors [6, 7]. With regard to driving, gender differences in aggression, risk-taking driving behavior, and noncompliance with regulations have been reported [8, 9]. From sociological and psychological perspectives, gender differences in these aspects have been attributed to sensation-seeking, dominance, and self-esteem/overconfidence [10–12]. Skaar and Williams [13] found that females characterized as busy, fast paced, and of high energy incurred more traffic violations, indicating that indeed male-assigned attributes increase noncompliance.

Contextual factors also play a role. For example, car types influence driving behavior. Regarding sport utility vehicles (SUV) and road safety, more risky traffic behavior has been observed, maybe due to the elevated sitting position and greater sense of security these vehicles provide in urban driving [14, 15]. On the other hand, it cannot be ruled out that individuals with a habit to take risks are more likely to drive a SUV. Choo et al. underlined that travel attitudes, personality, and lifestyle are important for vehicle type choice [16].

In the last two decades, a strong trend of increasing numbers of SUVs in streets has been noted in many countries. In Austria, their share increased from 8.2% in 2005 to approximately 23% (newly registered cars) in 2015 [17]. The rising number of SUVs and the subsequent effect on road safety has been recognized as an “emerging, and troubling, trend” and public health issue in recent years [18, 19].

It is known that SUVs generate a substantial risk for others. For each fatality avoided for an SUV or light-truck occupant, more than 4 fatalities are inflicted on others [20]. Furthermore, SUVs (as well as pickup trucks) were more often involved in pedestrian deaths, and upon accidents a higher pedestrian injury severity score was obtained [21–23]. In particular, light trucks and vans (including SUVs) were four times as likely to be associated with fatal injury of young children [24].

As some evidence showed a more deviant traffic behavior of SUV drivers [25], and the analyses by Ulfarsson and Mannering [14] revealed significant behavioral differences between male and female drivers, we were interested if there is a gender-specific SUV effect. Therefore, we conducted an observational study focusing on traffic violations and the gender of the driver in Vienna.

## Methods

### Setting and design

Three busy intersections in Vienna, Austria, were selected. The first site was a traffic hub in a mixed industrial and residential area. The second site was at a main arterial road in the inner city close to the University of Vienna, with heavy car and bicycle traffic as well as public transport and pedestrians. The third site was a residential area close to one high school and one elementary school. The sites were not in the vicinity of each other (minimum distance 3 km). Each site had good visibility of the carriageway.

### Collection of data

Observations were conducted on weekdays at the beginning of school term. We chose this time of the year (“back to school”) because road safety awareness campaigns are run by several institutions and in the media during this period. We picked that specific time period for a conservative approach, as at that time the issue of road safety is rather often focused in media. Furthermore, we wanted to avoid criticism that we selected a time period when less careful driving is expected. However, we did not expect that choice of the period would have an impact on a possible SUV effect.

Fifteen observers divided into teams of five recorded passing motor traffic at each location from Monday to Friday for one hour in the morning (7:30–8:30 a.m.), afternoon (1:00–2:00 p.m.), and early evening (4:00–5:00 p.m.). Altogether 45 sessions of observation were conducted within one week.

Before the observational phase had been started, observers attended a preparatory training by a specialist (mechanic) to learn to distinguish between specific car types. Only private passenger vehicles were included (excluding taxis, buses, delivery vans, and lorries that were not counted). Passenger vehicles were categorized into ordinary cars (defined as vehicles that are not designed to travel off road) and four wheel drive vehicles (4WD) and sport utility vehicles (SUV). For each eligible vehicle, the observer recorded its type (4WD/SUV or ordinary car), gender of the driver, and whether the driver was wearing a seat belt, using a handheld mobile phone while driving, and/or violating the traffic light. Furthermore, it was noted if a driver’s status could not be discerned (tinted windows or poor light).

Prior to the start of the study we conducted pretests with the observer teams at different busy intersections to guarantee consistent, uniform categorization.

In order to avoid double counting, each study assistant had to count vehicles of one lane and one category only. After the traffic light turned green the counting was written down in a protocol and the observation continued. If the vehicle could not be identified clearly, it was not added. However, our test runs showed that this occurred only rarely.

### Data analysis

We used SPSS statistical software (version 19, IBM Corp., USA) to analyze the data. For each site (1–3) and overall, each of the three violations were compared with respect to its frequency between drivers of ordinary passenger cars and SUVs with respect to their gender by logistic regression analysis. In particular, the probability p_ij_ to observe a violation in a person driving a specific type of car (i) being of gender (j) was modelled as: $\text{logit}\left (\mathrm{p}_{\text{ij}}\right )=\upalpha +\upbeta \cdot \updelta _{\mathrm{i}}+\upgamma \cdot \updelta _{\mathrm{j}}+\uplambda \cdot \updelta _{\mathrm{i}}\cdot \updelta _{\mathrm{j}}$.

In this equation, α, β, γ, and λ are the parameters estimated, δ_i_ is the indicator variable for SUV (it is 1 for an SUV driver and 0 otherwise), and δ_j_ is the indicator variable for gender (it is 1 for a female driver and 0 for a male driver). The exponential function, e. g., exp(β), of the parameters give the odds ratios. In addition, β is the parameter estimating the SUV effect, γ the parameter for female gender, and λ the parameter of interaction, i. e., the specific SUV effect in women. For each parameter estimate, the standard error is computed that allows testing the parameter against the zero hypothesis. For all tests, *p*-values below 0.05 were considered significant.

## Results

Altogether we counted 48,821 vehicles, of which 11.6% (*n* = 5653) were SUVs. This amounts to 1085 eligible vehicles, including 126 SUVs, per hour. A total of 25,809 vehicles (53%) were counted at the main arterial road in the inner city (site 2), 13,325 at the intersection in the residential/industrial area (site 1), and 9687 at the area close to a school (site 3). The percentage of female drivers among SUV drivers was 27% and close to the fraction of female drivers of other passenger vehicles (28%). Hence, the fraction of SUV drivers according to gender was similar (11.3% for females and 11.7% for males).

For all drivers together, 13.8% were not wearing seatbelts, 3.1% were using a handheld mobile phone while driving, and 2.5% violated traffic lights.

Traffic light violations were observed more frequently at site 2 (main arterial road), while driving unbelted and using a mobile phone occurred less frequently at this site. All violations (Table 1) were significantly more frequent in SUV drivers. Table 2 shows that male drivers were more likely to break traffic laws (16.1% driving unbelted, 3.1% using a mobile phone, 2.7% violating the traffic light) than female drivers (8.0% driving unbelted, 3.0% using a mobile phone, 1.9% violating the traffic light). The difference was statistically significant (*p* < 0.001) except for mobile phone use while driving.

**Table 1:** Total number of vehicles, number (percent) of sports utility vehicles (*SUV*) at the three crossroads and percentage of traffic violations (driving unbelted, mobile phone (*MP*) use without hands-free kit, red light violation) while driving a SUV or normal car

			Not belted (%)			MP use (%)			Red light (%)		
Place	Vehicles	SUV *n* (%)	SUV	Not SUV	*p*-value	SUV	Not SUV	*p*-value	SUV	Not SUV	*p*-value
1	13,325	1007 (7.6)	17.3	14.5	0.008	7.6	4.0	<0.001	3.7	1.7	<0.001
2	25,809	3402 (13.2)	15.0	13.0	0.001	4.3	2.4	<0.001	3.0	2.9	0.385
3	9687	1244 (12.8)	18.3	13.5	<0.001	4.9	2.4	<0.001	2.6	2.1	0.128
Total	48,821	5653 (11.6)	16.2	13.5	<0.001	5.0	2.9	<0.001	3.0	2.4	0.002

*p* -value comparing violations rates between SUV and normal cars

**Table 2:** Percentage of traffic violations (driving unbelted, mobile phone (*MP*) use without hands-free kit, red light violation) while driving a sports utility vehicle (*SUV*) or normal car according to gender

	Males			Females			Females overall		Females driving SUV	
Violation	SUV	Non SUV	Total	SUV	Non SUV	Total	OR (95% CI)	*p*-value	OR (95% CI)	*p*-value
Unbelted	18.1	15.8	16.1	10.9	7.7	8.0	0.44 (0.41–0.47)	<0.001	1.26 (1.04–1.52)	0.021
MP use	5.2	2.9	3.1	4.6	2.8	3.0	0.99 (0.87–1.12)	0.854	0.88 (0.65–1.20)	0.430
Red light	3.3	2.6	2.7	2.2	1.8	1.9	0.70 (0.60–0.81)	<0.001	0.95 (0.63–1.43)	0.805

Odds ratios (*OR*) for females and for interaction of females driving SUVs

*p* -value from logistic regression analysis

The largest gender difference was observed for driving without a seat belt – an offence that men are twice as likely to commit as women. This difference decreases if only SUVs are considered. For driving unbelted, the rate of traffic violations in women approaches that of males: The odds of driving without seat belts increased significantly for women driving SUVs by 26% (*p* = 0.021; Table 2). Only slight nonsignificant gender differences regarding use of a heldhand mobile phone while driving were registered (odds ratio = 0.99), whereby significant gender differences were found for violation of traffic lights (odds ratio = 0.7 for women; Table 2).

## Discussion

In Austria, talking on a handheld mobile phone while driving has been prohibited since 1999 and seat belts must be worn since 1976 [26, 27]. Nevertheless, we found a high level of noncompliance with these two important traffic laws in Vienna (driving unbelted: 14%, using a mobile phone: 3%). Traffic light violations were found in 2.5%. Considering that two violations are instantaneous (running a red light) or of short duration (using a mobile phone), the number of drivers violating these important traffic rules during a single journey must be considerable. Assuming that the probability of 2.5% of violating the traffic light is constant for all drivers and is equally and independently applicable to every crossroad with traffic lights the probability that at least one of 10 traffic lights is violated at one journey is 22%. For SUV drivers, this probability increases to 26%.

The comparison between SUVs (12% of the observed vehicles) and normal cars with respect to driver behavior shows clearly that drivers of SUVs were more likely to commit traffic law offences.

The “SUV effect” was highest for mobile phone use (for both women and men), followed by driving unbelted and violating traffic lights. The SUV effect in women was most pronounced for unbelted driving (in this case the effect was even stronger in women than in men).

The results – level of noncompliance regarding driving unbelted, mobile phone use – were comparable with those from London [25]. In this observational study, drivers of four wheel-drive vehicles were less likely to comply with the laws on using handheld mobile phones and on seat belts [25].

Consistent with previous research, we found a higher percentage of traffic violations among men [9, 28]. Interestingly, with regard to seat belt use the SUV effect impacts women more than men.

Our study was motivated by findings in London [25]. We considered gender to be an important aspect and included it in our design. We think that gender-tailored road-safety initiatives – if necessary – could be more effective. A “SUV effect” was found, in the sense that traffic violations are more often encountered in drivers of SUVs than in drivers of normal passenger cars. We found this effect in women for all violations and not significantly less pronounced than in men. However, for unbelted driving women showed an even stronger SUV effect than males.

How can these SUV effects be explained in general and especially in the case of women? Factors related to the car type might be the feeling of greater safety due to higher position of the driver and the altogether more sturdy nature of SUVs. It is known that women’s car buying is driven by different reasons (e. g., reliability and safety aspects) than men. The feeling of safety when driving a SUV might induce a more risky behavior especially in women. On the other hand, not wearing a seatbelt might make drivers steering more carefully. In that case, the accident risk might be reduced. Janssen [29] concluded that drivers were found to drive faster and less carefully when belted.

Effects of seat belt use on feelings of safety cannot be identified by an observational study like the present one. However, results regarding traffic light offenses indicate that SUV drivers do not drive more carefully.

As data from Germany indicate, SUV drivers are often executive staff or self-employed professionals [30]. This may be associated with tight work schedules that involve a great amount of mobility, communication, and coordination. Thus, this group is more likely to talk on the phone, and might be more likely to take this risk while driving because of the car’s characteristic and the feeling of safety.

For the first time, this study investigated gender aspects of the “SUV effect”. When driving a SUV, women approach frequency of certain violations of traffic law in males driving ordinary cars and regarding driving unbelted come even closer to the same behavior of men. As drivers of sport utility vehicles are more likely to show an unsafe driving behavior, this, on the one hand, adds to the risks of SUVs for other traffic participants [31]. On the other hand, it affects the safety of SUV drivers themselves. From a public health point of view, awareness campaigns specifically targeting drivers of SUVs and tailored also to female drivers should be designed and implemented. Further recommendations for specific traffic policy will depend on future research regarding the source of the SUV effect (driver’s personality or features of the SUV).

An observational study has intrinsic limitations. Most importantly, we cannot differentiate between cause and effect. That means we cannot exclude that attributes of the driver are the major factors responsible at the same time for choosing the car type and violating traffic laws. In this case, driving a SUV would be a coincidental factor. Whether the other explanation, that features of the car type, like feeling of safety, high sitting position etc., hence an original SUV effect, is the correct one, remains open for further research.

Discrimination between these different explanations is important for policy recommendations. It has been shown that safety measures and regulations could have the opposite effects than intended if behavioral consequences are neglected. If driving a SUV leads to more reckless driving, discouraging buying an SUV or focused road safety training for its users would be policy options. In contrast, if risk-taking behavior is the main factor checking such cars more frequently by the police would be a more appropriate response.

The same limitation holds for the specific ‘masculinizing’ effect we observed for driving unbelted in women SUV drivers. However, a behavioral effect is a more likely interpretation in this case.
